# Effects of Physical Exercise on Cardiorespiratory Fitness and Cardiometabolic Outcomes in Schizophrenia Spectrum Disorders: The FitForLife National Intervention in Sweden

**DOI:** 10.3390/life15101637

**Published:** 2025-10-21

**Authors:** Yvonne Forsell, Maria Skott, Buse Yel Bektash, Astrid Syvertsen, Örjan Ekblom, Catharina Lavebratt

**Affiliations:** 1 Department of Global Public Health, Karolinska Institutet, 171 77 Stockholm, Sweden; astrid.syvertsen@ki.se; 2 Department of Clinical Neuroscience, Karolinska Institutet, 171 77 Stockholmand Stockholm Health Care Services, 112 18 Stockholm, Sweden; maria.skott@regionstockholm.se; 3 Department of Molecular Medicine and Surgery, Karolinska Institutet, and Center for Molecular Medicine, Karolinska University Hospital, 171 76 Stockholm, Sweden; buse.yel.bektash@ki.se; 4 Division of Nursing, Department of Neurobiology, Care Sciences and Society, Karolinska Institutet, 141 83 Huddinge, Sweden; orjan.ekblom@ki.se; 5 Department of Physical Activity and Health, Swedish School of Sports and Health Sciences, 114 86 Stockholm, Sweden

**Keywords:** schizophrenia spectrum disorders, exercise intervention, cardiorespiratory fitness, cardiovascular risk factors

## Abstract

(1) Background: Individuals affected by schizophrenia spectrum disorders (SSDs) have an increased risk for cardiometabolic diseases. Improved cardiorespiratory fitness (CRF) is associated with lower cardiometabolic risk. The aim of the study was to analyze the effect of a six-month-long physical exercise intervention on CRF and cardiometabolic risk factors as well as whether the effect differed between sexes and different baseline CRF in SSD patients. (2) Methods: 122 patients at psychiatric open care units agreed to participate, 55 did not provide blood samples, and 14 dropped out, leaving 53 patients with complete pre–post data. BMI, waist–hip ratio, blood pressure, HbA1c, blood lipids, and CRF from ergometer bicycle tests were measured before and after the intervention. CRF was stratified into three groups. (3) Results: Cardiometabolic disturbances were common at baseline. After the intervention, all females and the group with the lowest CRF at baseline improved in triglyceride levels. The latter group also improved in CRF. (4) Conclusions: Females and those with the lowest baseline CRF had improved post-intervention, but causality cannot be inferred because our study was a non-randomized study without a control group.

## 1. Introduction

The disparity in life expectancy in individuals with schizophrenia spectrum disorders (SSD) remains wide; their life expectancy is 15–20 years shorter than the general population in Scandinavia [1]. This premature mortality in SSD is largely attributable to cardiometabolic diseases (CMD) [2,3], presenting as high rates of obesity, type 2 diabetes, and cardiovascular diseases [4,5,6].

Many factors contribute to the CMD risk in SSD, with antipsychotic drugs identified as a key driver [7]. Antipsychotic medications remain the cornerstone of the treatment of SSD and are necessary to prevent the psychotic relapses that define the disorder. However, they are associated with excessive weight gain, subsequently elevating the CMD risk. Nevertheless, modifiable factors also influence the CMD risk in SSD. For instance, individuals with SSD typically have sedentary lifestyles and exhibit high prevalence rates of tobacco and nicotine use [8,9,10]. Collectively, these factors appear to feed into a vicious cardiometabolic cycle, necessitating interventions that aim at modifying these CMD risk profiles.

It is well established that exercising regularly offers an array of beneficial effects, including reduced CMD risk, in the general population [11]. Specifically, medium- and high-intensity exercise elevating the heart rate enhances cardiorespiratory fitness (CRF), which is important for the body’s overall endurance. CRF is a measure of how well the cardiovascular and respiratory systems supply oxygen to the heart, lungs, and the circulatory system [12]. In the general population, improving CRF is associated with reducing the risk of cardiometabolic and cardiovascular events and reducing all-cause mortality [13,14].

Patients with schizophrenia, as a group, exhibit significantly lower CRF compared to age- and sex-matched controls, but the CRF varies between patients [15]. Considerable evidence also underscores that improving CRF, measured as the maximum volume of oxygen uptake (VO_2_max), in this patient group is possible [16]. To date, few intervention studies in SSD have evaluated the effect of physical exercise intervention across groups with different CRF at baseline. Understanding potential differential physical exercise treatment effects in SSD patients is crucial information when designing and tailoring exercise interventions in this heterogeneous patient group. The aim was to determine the pre–post change in CRF and cardiometabolic outcomes in a six-month-long exercise intervention study for persons affected with SSD in Sweden, led by SSD patients who were educated to be trainers. The pre–post change was investigated both in the total group, in males and females separately, and in groups stratified for CRF measured at baseline. We found that triglyceride levels were reduced in females, and that males and females with the lowest baseline CRF had improved triglyceride levels and CRF post-intervention, but causality cannot be inferred because our study was a non-randomized study without a control group.

## 2. Results

### 2.1. Participant Characteristics at Baseline and Intervention Adherence

**Figure 1:**
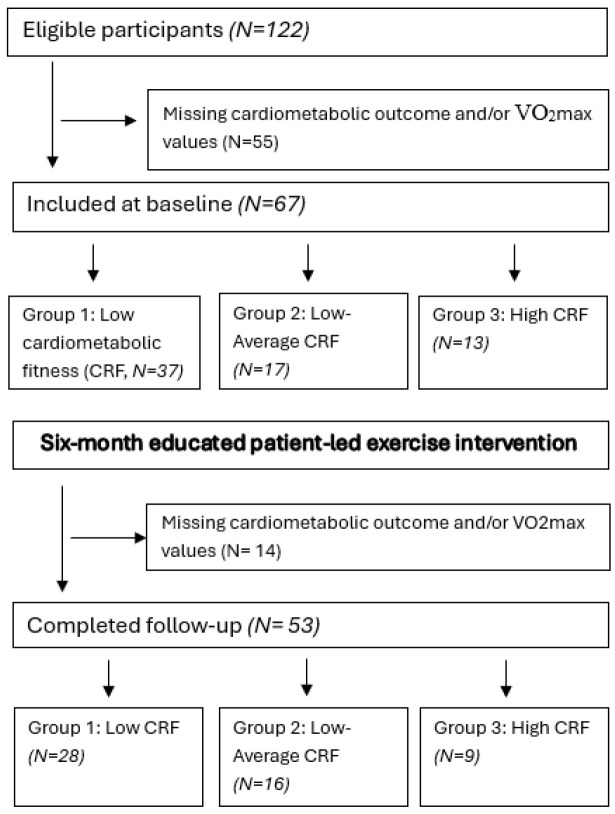
Flow chart of the study.

In total, 122 participants from 16 psychiatric units participated in the baseline examinations, but among these, 55 participants did not provide blood samples and/or VO_2_max at baseline (Figure 1). This yielded a study population of 67 participants. After the six-month intervention period, 14 participants were lost to follow-up, and hence, 53 (79.1%) participants also completed the 6-month intervention. An association was observed between baseline CRF level and exercise session attendance with participants in the high-CRF group (Group 3, N = 13) attending the most sessions (mean: 28.6, SD: 10.1), followed by the low–average CRF group (Group 2, N = 17) (24.7, SD: 10.3), and the low-CRF group (Group 1, N = 37) (18.0, SD: 10.9) (Table 1). An opposite trend was observed in the employment rate; those with the highest CRF had the lowest rate at baseline. Psychiatric service utilization was similar across groups, with the notable exception that all participants in the high-CRF group (100%) had SSD as their first F-diagnosis compared to 82.4% in the low–average group and 81.1% in the low-CRF group.

There also appeared to be a trend at baseline between CRF levels and cardiometabolic measures; those in the low-CRF group at baseline had the highest values in BMI, blood lipids, and Hb1Ac, followed by the low–average CRF group, and the high-CRF group, except for the Hb1Ac (Table 1). Overall, 82.1% of participants were overweight or obese (BMI ≥ 25 kg/m^2^), with obesity rates being highest in the low-CRF group (70.3% vs. 29.4% in low–average and 7.7% in high-CRF groups). Dyslipidemia was prevalent, with 73.1% of participants exhibiting elevated LDL/HDL ratios and 40.3% showing elevated triglyceride levels. These abnormalities were similarly common in the low-to-average CRF group, while the high-CRF group had the lowest prevalence of both. Participant characteristics and baseline cardiometabolic measures did not substantially differ between those lost to follow-up (N = 14) and those retained in the study (Supplementary Table S1).

### 2.2. Pre–Post Change in Cardiometabolic Outcomes

Sex-stratified analyses revealed significant improvements in triglyceride levels in females, a decrease of 0.42 mmol/L (95%CI: −0.71, −0.13; *p* = 0.0065), representing a clinically meaningful 23% reduction from baseline (Table 2). The triglyceride reduction in females showed a medium effect size (Cohen’s d = −0.56, Table S2). In contrast, there was an increase in BMI in males (0.55 kg/m^2^ (95%CI: 0.03, 1.07)), a small-to-medium effect (Cohen’s d = 0.44). Significant sex differences in pre–post changes were found for BMI (*p* = 0.037), while the other outcomes showed no significant sex differences (Table S2).

**Figure 2:**
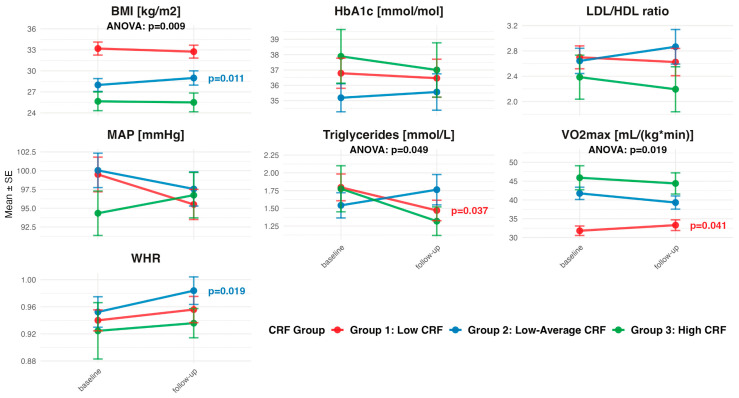
Pre–post changes observed in cardiometabolic outcome variables stratified by CRF grouping at baseline. For pre–post changes in outcome variables at *p* < 0.05, *p*-values are displayed next to the follow-up point. The ANOVA *p*-value is displayed for differences in change between CRF groups at *p* < 0.05. Bars represent mean ± SEM.

Participants with low baseline CRF (Group 1, N = 28) demonstrated the most favorable responses. There was a 1.49 mL/(kg ∗ min) increase in VO_2_max (95%CI: 0.07, 2.90), and −0.33 mmol/L reduction in triglyceride levels (95%CI: −0.63, −0.02) in the low-CRF group (Figure 2, Table S3). Comparisons between CRF-groups revealed significant differences for VO_2_max changes (*p* = 0.019) and triglyceride changes (*p* = 0.049). Those with low–average CRF (Group 2, N = 16) at baseline displayed a 0.03-unit increase in WHR (95%CI: 0.01, 0.06) and a 1.00 kg/m^2^ increase in BMI (95%CI: 0.26, 1.74) from baseline to follow-up. Differences between groups for BMI changes were significant (*p* = 0.009).

**Figure 3:**
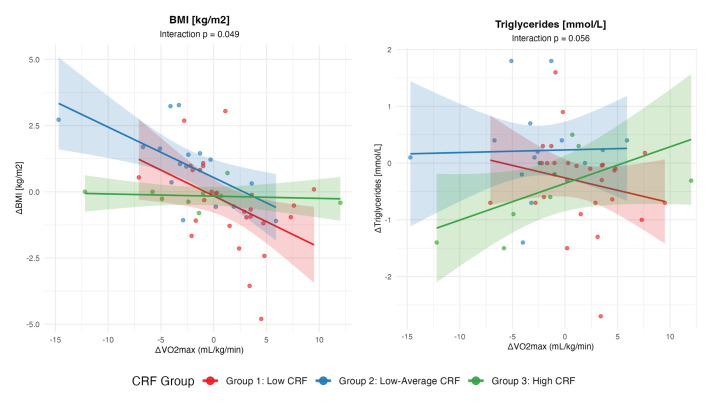
The effect per unit pre–post increase in VO_2_max on the pre–post change in BMI and plasma triglyceride levels, stratified by CRF grouping at baseline. Change (Δ) was calculated by subtracting baseline from follow-up values.

Linear regression analyses revealed that baseline fitness (CRF group) significantly moderated the relationship between CRF improvements (ΔVO_2_max) and BMI changes (interaction *p* = 0.049) (Figure 3, Table S4). The benefit of improved VO_2_max on BMI reduction was strongest in participants with lower baseline fitness (Group 1). For every 1 mL/kg/min improvement in VO_2_max, those with low baseline CRF experienced a 0.19 kg/m^2^ BMI reduction (95%CI: −0.33, −0.06; *p* = 0.0062). Participants with low–average baseline CRF (Group 2) showed similar benefits (−0.19 kg/m^2^; 95%CI: −0.34, −0.05; *p* = 0.011). In contrast, those with high baseline fitness (Group 3) showed no BMI benefit from VO_2_max improvements (β = −0.01, 95%CI: −0.15 to 0.13, *p* = 0.90). A similar pattern emerged for triglycerides, with a marginal interaction (*p* = 0.056) suggesting that participants with lower baseline fitness may derive greater triglyceride benefits from CRF improvements, though individual group effects did not reach statistical significance. No significant interactions with baseline fitness were observed for waist–hip ratio, mean arterial pressure, LDL/HDL ratio, or HbA1c (all *p* > 0.49), and VO_2_max improvements showed non-significant associations with these outcomes across all fitness groups.

## 3. Discussion

This 6-month exercise intervention, led by educated peers in individuals with SSD, demonstrated differential treatment responses based on baseline CRF levels. Participants with the lowest baseline CRF showed significant improvements in both fitness (VO_2_max) and triglyceride levels, while also demonstrating the strongest dose–response relationship between CRF gains and BMI reduction.

The high prevalence of cardiometabolic disturbances in our sample (82% overweight/obese, 77% with elevated WHR, and 40% with elevated triglyceride levels) aligns with the established literature documenting elevated cardiovascular risk factors in SSD populations [2,3]. The inverse relationship between baseline CRF and cardiometabolic risk factors supports previous findings that fitness disparities contribute significantly to health inequities in serious mental illness [15].

The triglyceride reduction found in females represented a 23% decrease from baseline, a substantial improvement that may translate to cardiovascular risk reduction. A meta-analysis of randomized trials shows that each 1 mmol/L reduction in triglyceride levels is associated with a 16% lower cardiovascular event risk [17].

The significant improvements in both CRF and triglyceride levels in the least fit group represent clinically meaningful changes. The CRF improvement (5% increase) aligns with previous meta-analyses showing 3–6% improvements in similar populations [16,18], while the triglyceride reduction (18% decrease) exceeds typical lifestyle intervention effects. However, the CRF improvement was quite small, which could be due to the fact that the exercise sessions only took place once a week and/or the low intensity of the sessions. The trainers were encouraged to increase the intensity over time and were coached by researchers from the Swedish School of Sport and Health Sciences. It was difficult to monitor the intensity during the sessions; our pre-study showed that using accelerometers was not feasible. Notably, similar to our study, some of the studies included in the aforementioned meta-analysis [16] were non-randomized, which severely limits causal inference.

The significant increases in WHR and BMI in the low–average CRF group were unexpected and warrant careful interpretation. Despite these anthropometric changes, this group showed non-significant trends toward improved metabolic markers, suggesting potential body composition changes rather than purely unfavorable effects. The significant interaction analysis indicates that if this group had achieved greater CRF improvements, they would have experienced BMI reductions like the low-CRF group. This suggests that the intervention intensity and/or frequency may have been suboptimal for this fitness level.

Participants with the highest baseline fitness showed minimal improvements and, contrary to expectations, demonstrated slight CRF decline. This group’s higher attendance rate compared to those with the lowest baseline fitness (29 vs. 18 sessions) suggests that motivation was not limiting, but rather that the intervention was suboptimal. This reflects well-documented ceiling effects where individuals with adequate fitness have limited capacity for improvement from moderate-intensity interventions.

The differential responses likely reflect varying physiological adaptation capacity across fitness levels. Less-fit individuals typically demonstrate greater plasticity in response to exercise training, experiencing larger improvements in insulin sensitivity, lipid metabolism, and cardiovascular efficiency [19]. The dose–response relationship observed in our interaction analysis supports this mechanism, suggesting that CRF improvements drive metabolic benefits in the less-fit individuals.

A recent systematic review and meta-analysis of exercise interventions in schizophrenia (17 studies) reported variable outcomes across studies [18]. Our findings extend this literature by demonstrating that treatment effects are substantially moderated by baseline fitness level. Also, our results help to explain the variability in previous findings. For instance, Fernández-Abascal et al. found no BMI changes but significant waist circumference improvements in a 12-week aerobic exercise intervention among SSD patients with metabolic syndrome, while our fitness-stratified analysis demonstrates that baseline CRF level significantly moderates metabolic treatment responses [20]. The mixed results across studies may partly reflect heterogeneous baseline fitness distributions in study populations.

The observed sex differences in exercise response may reflect distinct physiological adaptations between males and females. The triglyceride reduction observed specifically in females aligns with evidence suggesting that women may exhibit greater metabolic improvements from aerobic exercise interventions [21]. Conversely, the BMI increase in males could be attributed to exercise-induced muscle hypertrophy, as males typically demonstrate greater capacity for skeletal muscle mass gains compared to females. Since BMI does not distinguish between muscle and fat mass, increases in lean body mass from resistance or combined training components could manifest as BMI elevation despite favorable body composition changes.

Our findings support baseline fitness assessments to guide exercise prescription intensity and frequency in SSD populations. The peer-led intervention model demonstrated feasibility across fitness levels, with attendance rates (18–29 sessions over 6 months) exceeding those reported in many supervised exercise trials [17]. The model offers opportunities for sustainable implementation despite limited healthcare resources, with peer trainers serving as role models to enhance engagement.

Based on these findings, we recommend prioritizing less-fit individuals for group-based exercise interventions and considering fitness level when setting realistic treatment expectations and outcomes. Fitter patients might need help to utilize other physical exercise options outside of the clinic.

### Strengths and Limitations

Strengths include the use of validated submaximal fitness testing suitable for clinical populations, an innovative peer-led intervention model enhancing sustainability, comprehensive cardiometabolic assessment, and novel fitness-stratified analysis addressing a significant literature gap. The study achieved good retention (79%) and balanced sex representation, which is notable given reported male reluctance to participate in exercise interventions [22].

Several limitations should be acknowledged. The modest sample size limits the statistical power for subgroup analyses and controls for psychiatric medications that could influence cardiometabolic outcomes. Random errors from variations in the CRF testing procedure, by being performed at 16 clinics, increase the risk of not detecting true change. We were unable to standardize the fasting state at blood sampling due to participants’ varied diurnal rhythms. The post hoc CRF stratification limits causal inference about differential treatment effects. Additionally, we had limited data on baseline illness severity beyond participants being in remission and receiving open psychiatric care, creating potential unmeasured confounding from heterogeneous symptom severity and treatment history. Finally, the 6-month follow-up may be insufficient to assess the long-term sustainability of benefits. The single-arm intervention design severely limits causal inference. Randomized controlled trial (RCT) design on an individual level was not used due to the following: (i) the small size of the clinical units, 25–100 patients with SSD each; (ii) patients within a unit meeting in another type of group session; and (iii) ethical concerns if the exercise intervention was not offered to all patients; and it was practically impossible with a waiting list. Adequate randomization at the unit level was unachievable because of the different unit sizes and different staff turn-over rates. Priority areas for future investigation include randomized stratified designs comparing fitness-tailored versus standard interventions to establish causal relationships, mechanistic studies examining physiological pathways underlying differential treatment responses, and biomarker studies investigating inflammatory and metabolic mediators of exercise response.

## 4. Materials and Methods

### 4.1. Study Setting

The data was retrieved from the FitForLife national intervention study, aiming at improving cognition, overall wellbeing, and psychosocial function, as well as cardiometabolic outcomes and CMD risk in SSD patients. The FitForLife national intervention study was a six-month-long group-based exercise intervention led by SSD patients who were educated to be trainers. The study was carried out at 16 outpatient psychiatric units specialized in care for individuals affected by SSD between 2021 and 2023. The included units were in the regions of Stockholm, Skåne, and Halland in Sweden, covering approximately 40% of the Swedish population [23]. Before the intervention, the only exercise offered at these clinics was weekly walking groups at some units. Within the six months of the FitForLife intervention, all units offered at least one exercise session per week.

### 4.2. Recruitment of Study Participants

All participants at the units were invited to participate in the study, and all interested participants had an initial clinical evaluation by their clinical care provider. Exclusion criteria at baseline were acute psychosis and overall somatic conditions that could be aggravated by physical exercise. The participants were assessed with examinations and interviews at baseline and follow-up by qualified staff who were educated by the researchers. Prior to inclusion, informed consent was collected from all participants. The FitForLife national trial was approved by the Regional Ethics Review Board in Stockholm: 2019-03409 and amendments. The trial is registered in ClinicalTrials.Gov with the ID number: NCT04239612.

### 4.3. Exercise Regimen

The group-based exercise sessions took place at the outpatient units. Every exercise session started with 5–10 min of warm-up and was followed by 45 min of high-intensity circuit-based strength training using one’s own body weight. Finally, each exercise session was completed with 5–10 min of stretching. The design of the exercise regimen derives from the guidelines formulated by the American College of Sports Medicine for prescribing exercise, and adjustments were made by the exercise leaders in collaboration with the Swedish School of Sport and Medicine (GIH) [24].

### 4.4. Examinations and Interviews of Participants

At baseline and after the 6 months of intervention (at follow-up), each participant was interviewed by a nurse at the clinical unit. Characteristics collected included sex (male or female), age, occupational status, the cumulative number of attended exercise sessions, prescribed antipsychotic medication (ATC: NO5A, all generations), and the cumulative number of inpatient psychiatric care hospitalizations. Even though most of the participants held an SSD diagnosis, many had received multiple psychiatric disorder diagnoses since their first contact with psychiatric care. Therefore, we also retrieved the proportion of participants who received an SSD diagnosis as their first F-diagnosis. The following cardiometabolic measures were also collected through examination and blood sampling [cut-offs for pathological levels]: Waist–hip circumference ratio (WHR) [females ≥0.85, males ≥0.90] [25], body mass index (BMI) [overweight: 25.0–29.9 kg/m^2^, obesity: 30.0–39.9 kg/m^2^, severe obesity: ≥40 kg/m^2^] [26], mean atrial blood pressure (MAP) = diastolic blood pressure + 1/3(systolic blood pressure − diastolic blood pressure) [≥100 mmHg] [27], HbA1c [≥42 mmol/mol] [28], LDL/HDL ratio [≥2.0 for elevated levels] [29], and triglycerides [≥1.7 mmol/L for borderline high levels] [30]. The blood markers were analyzed by accredited clinical chemistry laboratories using similar analytical platforms.

CRF, expressed as maximum oxygen uptake (VO_2_max), was determined using the Ekblom-Bak (EB) ergometer cycle test at baseline and follow-up [31]. It is a submaximal test, making it suitable to assess participants who may be unwilling or unable to complete a standard VO_2_max test. The EB test consists of two workloads, each 4 min long, and the difference in heart rate between them is used to calculate the participants’ VO_2_max. During the first workload, the participant bikes on a workload that is standard for all participants while keeping a pedaling frequency of 60 revolutions per minute [31,32]. The heart rate is noted during the fourth minute, and the average is used for calculation. Immediately moving on to the second workload, a higher load is set based on preselected criteria (BMI, age, and sex), aiming for a perceived exertion value of ~14, based on the Borg’s scale (a perceived scale from 6 to 20, where the value of 6 implies no exhaustion and 20 implies exertion and pain) [33]. Again, the average heart rate during the fourth minute is noted. The EB test calculates the VO_2_max based on changes in heart rate between two sets of submaximal ergometer workloads, yielding (i) absolute VO_2_max (unit L/min), which represents the total and maximum amount of oxygen supplied to the body, and (ii) relative VO_2_max (unit mL/(kg ∗ min)), which takes the person’s body weight into account. Given the heterogeneity in this study population, we employed the relative VO_2_max measure. Relative VO_2_max at baseline and follow-up was calculated using age, body weight, standard heart rate during the first workload, higher heart rate during the second workload, and the predetermined factor for higher workload [34]. CRF test staff were not the same individuals at all clinics, but all were trained by the same teacher for this specific testing procedure. For almost all patients, the same test staff performed pre- and post-measurements.

### 4.5. Statistical Analysis

Pre-to-post intervention changes in CRF and cardiometabolic outcomes were calculated by subtracting baseline from follow-up values. Complete case analysis was performed for each outcome variable. Normality of change distributions was assessed using the Shapiro–Wilk tests. Paired sample *t*-tests were performed to evaluate pre–post changes for the total sample and stratified by sex, as *t*-tests are robust to departures from normality with moderate sample sizes. Results are reported as mean change with 95% confidence intervals. Statistical significance was set at two-tailed *p* < 0.05.

Participants were stratified according to baseline CRF using age- and sex-specific reference values for VO_2_max from the ergometer bicycle test in the Swedish working population [35]. The reference values by Väisänen et al. yield seven CRF categories: “very low”, “low”, “somewhat low”, “average”, “somewhat high”, “high”, and “very high”. Due to statistical power considerations and the baseline distribution of VO_2_max in our sample, CRF categories were collapsed into three groups: low CRF (combining “very low” and “low”), low–average CRF (combining “somewhat low” and “average”), and high CRF (combining “somewhat high”, “high”, and “very high”) (Table S5).

Pre–post change analyses were performed within each CRF category using paired sample *t*-tests. Mean changes and 95% CIs were calculated for VO_2_max and all cardiometabolic outcomes within each CRF group. Differences between groups in pre–post changes across the three baseline CRF groups were tested using one-way analysis of variance (ANOVA). A between-group *p*-value < 0.05 was considered statistically significant.

Linear regression models with interaction terms were used to investigate whether the effect of VO_2_max improvements on cardiometabolic outcomes differed by baseline CRF level. The model specification was Outcome ~ ΔVO_2_max × CRF_group, where ΔVO_2_max represents the pre–post change in VO_2_max (mL/kg/min), CRF_group represents baseline fitness categories (Group1 as reference), and the interaction term (ΔVO_2_max × CRF_group) tests whether the association between VO_2_max improvement and outcome changes differs across fitness groups.

Interaction significance was evaluated using F-tests comparing nested models with and without interaction terms. Group-specific effects were estimated using estimated marginal trends (emtrends) from the emmeans package, which calculates the slope of VO_2_max change for each CRF group while accounting for the interaction structure. Results are presented as regression coefficients representing the change in each outcome per 1 mL/kg/min improvement in VO_2_max for each CRF group, with 95% confidence intervals. Statistical significance was set at *p* < 0.05.

Model assumptions were assessed through visual inspection of diagnostic plots, including residuals versus fitted values plots (linearity and homoscedasticity), Q-Q plots of residuals (normality of residuals), and Cook’s distance plots (influential observations). Normality of residuals was tested using the Shapiro–Wilk tests, and homoscedasticity was assessed using Breusch–Pagan tests when available. Complete case analysis was performed for each outcome variable. All statistical analyses were conducted using R version 4.5.1 (R Foundation for Statistical Computing, Vienna, Austria) with packages dplyr, broom, emmeans, lmtest, and ggplot2.

## 5. Conclusions

In this physical exercise intervention among individuals with SSD, females and those with the lowest baseline CRF had the greatest cardiometabolic benefits after peer-led group training. However, as it was not an RCT, causality cannot be inferred. The significant improvements in fitness and triglycerides in this low-CRF subgroup, coupled with dose–response evidence from previous RCTs, propose fitness-stratified exercise prescription in clinical practice. However, the unexpected unfavorable changes in the low–average fitness group highlight the need for careful intervention tailoring. The findings need to be confirmed with an RCT.

## Figures and Tables

**Table 1:** Participant characteristics at baseline stratified by cardiorespiratory fitness (CRF) categories.

Characteristic	Group 1 Low CRF (N = 37)	Group 2 Low–Average CRF (N = 17)	Group 3 High CRF (N = 13)	Total (N = 67)
Male sex; %	46.0	58.8	46.2	49.3
Age; Mean (S.D.)	44.6 (10.2)	47.5 (11.2)	50.5 (9.1)	46.5 (10.4)
Employed or student; % *	21.6	23.5	15.4	20.9
Number of exercise sessions in clinic; Mean (S.D.)	18.0 (10.9)	24.7 (10.3)	28.6 (10.1)	21.7 (11.3)
**Psychiatric service utilization**				
SSD as first ICD-10 F-diagnosis; % **	81.1	82.4	100.0	85.1
Number of times inpatient; Mean (S.D.)	3.0 (2.5)	5.3 (3.3)	3.9 (3.0)	3.7 (2.9)
Current antipsychotic prescription; %	89.2	94.1	84.6	89.6
**Cardiometabolic risk factors**				
WHR; Mean (S.D.)	0.93 (0.09)	0.95 (0.09)	0.93 (0.11)	0.94 (0.09)
Normal (♂ ≤ 0.89, ♀ ≤ 0.84); %	24.32	11.76	30.77	22.39
Elevated (♂ ≥ 0.90, ♀ ≥ 0.85); %	75.68	88.24	69.23	77.61
BMI [kg/m^2^]; Mean (S.D.)	32.39 (5.53)	28.03 (3.56)	25.43 (3.50)	29.93 (5.51)
Normal (≤24.9); %	8.1	17.7	46.2	17.9
Overweight (25.0–29.9); %	21.6	52.9	46.2	34.3
Obese (30–39.9); %	59.5	29.4	7.7	41.8
Severely obese (≥40.0) %	10.8	0.0	0.0	6.0
MAP blood pressure [mmHg]; Mean (S.D.)	98.0 (11.9)	100.1 (8.9)	93.9 (11.5)	97.8 (11.2)
Normal MAP (60.9–99.9); %	56.8	47.1	53.9	53.7
High MAP (≥100.00); %	43.2	52.9	46.2	46.3
Triglycerides [mmol/L]; Mean (S.D.)	1.75 (1.03)	1.56 (0.69)	1.52 (0.9)	1.66 (0.93)
Normal (≤1.6); %	59.5	52.9	69.2	59.7
Elevated (≥1.7); %	40.5	47.1	30.8	40.3
LDL/HDL ratio; Mean (S.D)	2.72 (0.91)	2.63 (0.77)	2.24 (0.96)	2.61 (0.9)
Normal (<2); %	21.6	23.5	46.2	26.9
Elevated (≥2.0); %	78.4	76.5	53.9	73.1
HbA1c [mmol/mol]; Mean (S.D.)	36.4 (4.8)	35.4 (3.7)	36.2 (5.3)	36.1 (4.6)
Normal (≤41); %	86.5	94.1	92.3	89.6
Pre-diabetes and diabetes (≥42); %	13.5	5.9	7.7	10.5

* Data on occupational status was missing from 12 participants. ** Other ICD-10 F-diagnosis: Asperger’s syndrome; other specified organic personality and behavioral disorders due to brain disease, brain damage, or cerebral dysfunction; severe depressive episode with psychotic symptoms; unspecified organic or symptomatic mental disorder; bipolar disorder or manic episode with psychotic symptoms; recurrent depression or a severe episode with psychotic symptoms; hyperactivity disorder, unspecified; panic disorder [episodic paroxysmal anxiety]; lasting personality change as a result of disaster experience; and mania without psychotic symptoms. ICD-10 = 10th revision of the International Classification of Diseases, WHR = waist–hip ratio, BMI = body mass index, MAP = mean arterial pressure, LDL = low-density cholesterol; HDL = high-density cholesterol, and HbA1c = Glycosylated hemoglobin.

**Table 2:** Pre–post change in cardiorespiratory fitness (CRF) and other cardiometabolic outcomes were analyzed using paired *t*-tests in total sample and stratified by gender.

Outcome Variable (N = 53)	Baseline: Mean (S.D.)	Follow-Up: Mean (S.D.)	Pre–Post Change: Mean (95%CI)
VO_2_max [mL/(kg ∗ min)]	37.2 (9.2)	37.0 (8.5)	−0.2 (−1.5, 1.1)
WHR	0.94 (0.09)	0.96 (0.09)	0.02 (0.00, 0.04)
BMI [kg/m^2^]	30.34 (5.37)	30.38 (5.23)	0.04 (−0.39, 0.47)
MAP [mmHg]	98.8 (10.9)	96.3 (9.8)	−2.5 (−5.8, 0.9)
Triglycerides [mmol/L]	1.72 (0.91)	1.53 (0.77)	−0.18 (−0.40, 0.04)
Ldl/Hdl ratio	2.63 (0.92)	2.62 (1.11)	0.00 (−0.19, 0.18)
HbA1c [mmol/mol]	36.5 (4.8)	36.3 (5.8)	−0.21 (−1.11, 0.65)
**Males (**N **= 25)**			
VO_2_max [mL/(kg ∗ min)]	42.9 (6.7)	41.7 (7.5)	−1.6 (−3.4, 1.1)
WHR	0.99 (0.08)	1.00 (0.07)	0.01 (0.00, 0.03)
BMI [kg/m^2^]	29.66 (4.77)	30.21 (4.94)	**0.55 (0.03, 1.07)**
MAP [mmHg]	98.9 (11.0)	98.1 (7.7)	−0.8 (−5.6, 3.9)
Triglycerides [mmol/L]	1.63 (0.78)	1.71 (0.95)	0.08 (−0.24, 0.41)
Ldl/Hdl ratio	2.91 (0.68)	3.01 (1.19)	0.10 (−0.25, 0.44)
HbA1c [mmol/mol]	35.5 (3.5)	35.6 (4.1)	0.12 (−0.85, 1.09)
**Females (**N **= 28)**			
VO_2_max [mL/(kg ∗ min)]	32.1 (8.2)	32.8 (7.1)	0.6 (−1.0, 2.2)
WHR	0.9 (0.08)	0.92 (0.09)	0.03 (−0.02, 0.07)
BMI [kg/m^2^]	30.95 (5.87)	30.54 (5.57)	−0.41 (−1.06, 0.23)
MAP [mmHg]	98.7 (10.9)	94.8 (11.3)	−3.9 (−8.9, 1.1)
Triglycerides [mmol/L]	1.79 (1.01)	1.37 (0.53)	**−0.42 (−0.71, −0.13)**
Ldl/Hdl ratio	2.38 (1.03)	2.28 (0.93)	−0.10 (−0.29, 0.10)
Hba1c [mmol/mol]	37.4 (5.6)	36.9 (7.0)	−0.50 (−1.93, 0.93)

Bold: Pre-Post Change denotes statistically significant pre–post change. VO_2_max = relative submaximal oxygen intake volume per body weight and minute as a measure of CRF.

## Data Availability

The data presented in this study are available on request from the corresponding author due to ethical restrictions.

## Supplementary Materials

The following supporting information can be downloaded at https://www.mdpi.com/article/10.3390/life15101637/s1; Table S1: Participant characteristics at baseline, stratified by those lost to follow-up and retained participants; Table S2: Effect sizes (Cohen’s d) for pre–post changes in VO_2_max and other cardiometabolic outcomes in total sample, only males and only females; Table S3: Pre–post change in outcome variables stratified by cardiorespiratory fitness (CRF) groups at baseline; Table S4: The effect of VO_2_max change on outcome variables, stratified by baseline cardiorespiratory fitness (CRF) groups, assessed using linear regression; Table S5: Baseline VO_2_max limits of the cardiorespiratory fitness (CRF) groups by sex and age.

## Abbreviations

The following abbreviations are used in this manuscript:

TLA	Three-letter acronym
SSD	Schizophrenia spectrum disorders
CRF	Cardiorespiratory fitness
CMD	Cardiometabolic diseases
VO2max	Volume of oxygen uptake
ICD-10	10th revision of the International Classification of Diseases
BMI	Body mass index
WHR	Waist–hip circumference ratio
MAP	Mean arterial pressure
LDL	Low-density cholesterol
HDL	High-density cholesterol
GIH	Swedish School of Sport and Medicine
