# Supplementary material for: Effects of Physical Exercise on Cardiorespiratory Fitness and Cardiometabolic Outcomes in Schizophrenia Spectrum Disorders: The FitForLife National Intervention in Sweden
Source: Life (Basel). 2025 Oct 21;15(10):1637. doi: 10.3390/life15101637 (PMC12565665; doi:10.3390/life15101637)
Supplement: Supplementary file 1 [file life-15-01637-s001.zip › life-3853852-supplementary.pdf]

Supplementary Information

Effects of physical exercise on cardiorespiratory fitness and cardiometabolic outcomes in Schizophrenia Spectrum Disorders: The FitForLife national intervention in Sweden.

Yvonne Forsell[1], Maria Skott[2], Buse Yel Bektash[3], Astrid Syvertsen[1], Örjan Ekblom[4,5], Catharina Lavebratt[3]

Supplementary Table S1. Participant characteristics at baseline, stratified by lost to follow-up and retained participants.

Supplementary Table S2. Effect sizes (Cohen's d) for pre-post changes in VO_2max and other cardiometabolic outcomes in total sample, only males and only females.

Supplementary Table S3. Pre-post change in outcome variables stratified by cardiorespiratory fitness (CRF) group at baseline.

Supplementary Table S4. The effect of VO_2max change on outcome variables, stratified by baseline cardiorespiratory fitness (CRF) groups, assessed using linear regression.

Supplementary Table S5. Baseline VO_2max limits of the cardiorespiratory fitness (CRF) groups by sex and age.

Table S1. Participant characteristics at baseline, stratified by lost to follow-up and retained participants.

Characteristic	Lost to follow-up (N=14)	Retained (N=53)	p-value
Male sex (%)	57.1	47.2	0.72
Age; Mean (S.D.)	43.4 (10.2)	47.3 (10.4)	0.20
Employed or student (%)*	21.4	20.8	1.0
Number of exercise sessions in clinic; Mean (S.D.)	22.1 (16.9)	21.6 (9.4)	0.83
Psychiatric service utilization			
SSD as first ICD-10 F-diagnosis; %	92.9	83.0	0.67
Number of times inpatient; Mean (S.D.)	3.8 (2.8)	3.7 (3.0)	0.78
Current antipsychotic prescription; %	100.0	86.8	0.33
Cardiometabolic risk factors			
WHR; Mean (SD)	0.93 (0.10)	0.94 (0.09)	0.12
Normal (♂≤0.89, ♀≤0.84); %	28.57	20.75	0.50
Elevated (♂≥0.90, ♀≥0.85); %	71.43	79.25	
BMI [kg/m^2]; Mean (S.D.)	28.4 (6.0)	30.3 (5.4)	0.12
Normal (≤24.9); %	28.6	15.1	
Overweight (25.0-29.9); %	42.9	32.1	
Obese (30-39.9); %	21.4	47.2	0.14
Severely obese (≥40.0) %	7.14	5.7	
MAP blood pressure [mmHg] ; Mean (S.D.)	93.8 (11.9)	98.8 (10.9)	0.48
Normal MAP (60.9-99.9); %	57.1	52.8	
High MAP (≥100.00); %	42.9	47.2	1.0
Triglycerides [mmol/L]; Mean (S.D.)	1.45 (1.01)	1.72 (0.91)	0.16
Normal (≤1.6); %	78.6	54.72	
Elevated (≥1.7); %	21.4	45.28	0.13
LDL/HDL ratio; Mean (S.D)	2.52 (0.84)	2.63 (0.92)	0.80
Normal (< 2); %	21.4	28.3	
Elevated (≥ 2.0); %	78.6	71.7	0.74
HbA1c [mmol/mol]; Mean (S.D.)	34.71 (3.36)	36.49 (4.8)	
Normal (≤41); %	100.0	86.8	
Pre-diabetes & diabetes (≥42); %	0	13.2	0.33

*Data on occupational status was missing from 12 participants

Table S2. Effect sizes (Cohen's d) for pre-post changes in VO_2max and other cardiometabolic outcomes in total sample, only males and only females.

**Outcome variable**	**Cohen's d (Total)**	**Cohen's d (Males)**	**Cohen's d (Females)**	**Sex difference p-value**
VO_2max [mL/(kg*min)]	0.044	-0.21	-0.16	0.19
WHR	0.23	0.30	0.24	0.66
BMI [kg/m^2]	0.027	0.44	-0.25	0.020
MAP [mmHg]	-0.20	-0.073	-0.30	0.48
Triglycerides [mmol/L]	-0.23	0.11	-0.56	0.051
Ldl/Hdl ratio	-0.0069	0.12	-0.19	0.32
HbA1c [mmol/mol]	-0.067	0.051	-0.14	0.28

Table S3. Pre-post change in outcome variables stratified by cardiorespiratory fitness (CRF) group at baseline.

**Outcome variable**	**Group 1: Low CRF** Mean (95% CI) (N=28)	**Group 2: Low-Average CRF** Mean (95% CI) (N=16)	**Group 3: High CRF** Mean (95%CI) (N=9)	**p-value**
VO_2max [mL/(kg*min)]	**1.49 (0.07, 2.90)**	-2.44 (-4.90, 0.02)	-1.52 (-6.54, 3.49)	0.019
WHR	0.02 (-0.03, 0.01)	**0.03 (0.01, 0.06)**	0.01 (-0.05, 0.07)	0.81
BMI [kg/m^2]	-0.44 (-1.08, 0.19)	**1.00 (0.26, 1.74)**	-0.15 (-0.47, 0.16)	0.0085
MAP [mmHg]	-4.00 (-9.48, 1.48)	-2.48 (-7.47, 2.51)	2.4 (-4.99, 9.80)	0.40
Triglycerides [mmol/l]	**-0.33 (-0.63, -0.02)**	0.22 (-0.20, 0.64)	-0.46 (-1.00, 0.09)	0.049
Ldl/Hdl ratio	-0.08 (-0.40, 0.22)	0.22 (-0.08, 0.53)	-0.19 (-0.64, 0.25)	0.25
Hba1c [mmol/mol]	-0.32 (-1.69, 1.05)	0.38 (-1.19, 1.94)	-0.89 (-2.35, 0.57)	0.61

Bold denotes statistically significant mean pre-post change in the outcome variable. The p-value is derived from comparing the pre-post change between the three CRF groups.

Table S4. The effect of VO_2max change on outcome variables, stratified by baseline cardiorespiratory fitness (CRF) groups, assessed using linear regression.

Model used for group-stratified analyses: Outcome = $\beta_0 + \beta_1(VO_2max\_change) + \beta_2(CRF\_group2) + \beta_3(CRF\_group3) + \beta_4(VO_2max\_change \times CRF\_group2) + \beta_5(VO_2max\_change \times CRF\_group3) + \varepsilon$

Model coefficients:

- **β_0** = Intercept for reference group (Group1)
- **β_1** = Slope of VO_2max_change for reference group (Group1)
- **β_2, β_3** = Difference in intercepts for Group2 and Group3 vs Group1
- **β_4, β_5** = **Interaction terms -** difference in slopes for Group2 and Group3 vs Group1
- **CRF_group** is factored with levels: Group1 (reference), Group2, Group3

Note: Group-specific values shown below represent total slopes: Group1 = β_1; Group2 = $\beta_1 + \beta_4$; Group3 = $\beta_1 + \beta_5$

**Outcome variable**	**Entire sample** (95% CI) (N=53)	**Group 1** **Low CRF**; (95%CI) (N= 28)	**Group 2** **Low-Average CRF**; (95% CI) (N= 16)	**Group 3** **High CRF**; (95% CI) (N= 9)	**p-value of interaction***
WHR	-0.00 (-0.01, 0.00)	-0.00 (-0.01, 0.01)	0.00 (-0.01, 0.01)	0.00 (-0.01, 0.01)	0.84
BMI [kg/m^2]	**-0.16 (-0.24, -0.08)**	**-0.19 (-0.33, -0.06)**	**-0.19 (-0.34, -0.05)**	-0.01 (-0.15, 0.13)	0.049
MAP [mmHg]	-0.50 (-1.20, 0.20)	-0.91 (-2.21, 0.39)	-0.05 (-1.33, 1.43)	-0.47 (-1.81, 0.87)	0.68
Triglycerides [mmol/L]	-0.01 (-0.06, 0.04)	-0.04 (-0.13, 0.04)	0.00 (-0.12, 0.03)	0.06 (-0.02, 0.15)	0.056
Ldl/Hdl ratio	-0.03 (-0.07, 0.01)	-0.05 (-0.012, 0.02)	-0.04 (-0.12, 0.03)	0.01 (-0.06, 0.08)	0.49
Hba1c [mmol/mol]	-0.03 (-0.21, 0.16)	-0.02 (-0.36, 0.33)	-0.02 (-0.38, 0.35)	-0.01 (-0.36, 0.35)	0.93

Bold denotes statistically significant association between VO_2max change and outcome variable change ($p<0.05$) assessed with individual t-tests.

*Interaction p-values calculated using F-test comparing models with and without interaction terms.

Table S5. Baseline VO_2max limits of the cardiorespiratory fitness (CRF) groups by sex and age.

**Sex**	**Age** (years)	**Lowest VO_2max in Group 2: Low-Average** (mL/(kg*min)	**Lowest VO_2max in Group 3: High** (mL/(kg*min)
Males	20-24	45	53
	25-29	43	51
	30-34	41	49
	35-39	40	47
	40-44	38	45
	45-49	37	43
	50-54	35	41
	55-59	33	38
	60-64	32	37
	65+	31	35
Females	20-24	35	42
	25-29	35	42
	30-34	33	40
	35-39	33	39
	40-44	31	38
	45-49	30	36
	50-54	29	35
	55-59	28	33
	60-64	27	32
	65+	26	32

Thresholds were obtained from Väisänen et al 2024.

Väisänen, D.; Ekblom, B.; Wallin, P.; Andersson, G.; & Ekblom-Bak, E. Reference values for estimated VO2max by two submaximal cycle tests: The Åstrand-test and the Ekblom-Bak test. *European Journal of Applied Physiology*, **2024,** *124(6),* 1747-1756. https://doi.org/10.1007/s00421-023-05398-8
